# A Novel Planar Grounded Capacitively Coupled Contactless Conductivity Detector for Microchip Electrophoresis

**DOI:** 10.3390/mi13030394

**Published:** 2022-02-28

**Authors:** Jianjiao Wang, Yaping Liu, Wenhe He, Yuanfen Chen, Hui You

**Affiliations:** 1 School of Mechanical Engineering, Guangxi University, Nanning 10593, China; 1811301028@st.gxu.edu.cn (J.W.); 2011391061@st.gxu.edu.cn (Y.L.); yuanfenchen@gxu.edu.cn (Y.C.); 2 School of Electrical Engineering, Guangxi University, Nanning 10593, China; 1812401002@st.gxu.edu.cn

**Keywords:** microchip electrophoresis, stray capacitance, capacitively coupled contactless conductivity detection, planar grounded electrode

## Abstract

In the microchip electrophoresis with capacitively coupled contactless conductivity detection, the stray capacitance of the detector causes high background noise, which seriously affects the sensitivity and stability of the detection system. To reduce the effect, a novel design of planar grounded capacitively coupled contactless conductivity detector (PG-C4D) based on printed circuit board (PCB) is proposed. The entire circuit plane except the sensing electrodes is covered by the ground electrode, greatly reducing the stray capacitance. The efficacy of the design has been verified by the electrical field simulation and the electrophoresis detection experiments of inorganic ions. The baseline intensity of the PG-C4D was less than 1/6 of that of the traditional C4D. The PG-C4D with the new design also demonstrated a good repeatability of migration time, peak area, and peak height (*n* = 5, relative standard deviation, RSD ≤ 0.3%, 3%, and 4%, respectively), and good linear coefficients within the range of 0.05–0.75 mM (R^2^ ≥ 0.986). The detection sensitivity of K^+^, Na^+^, and Li^+^ reached 0.05, 0.1, and 0.1 mM respectively. Those results prove that the new design is an effective and economical approach which can improve sensitivity and repeatability of a PCB based PG-C4D, which indicate a great application potential in agricultural and environmental monitoring.

## 1. Introduction

Microchip electrophoresis has the characteristics of small size (~cm^2^) and short separation channels, which is efficient and convenient for on-site and real-time detection. Combined with C4D, microchip electrophoresis device is easy to miniaturize, avoiding solution contamination, as well as large and precise instrument requirement. Thus, C4D technology has been widely applied in capillary electrophoresis and microchip electrophoresis [1,2,3,4].

C4D was first proposed independently by Zemann et al. [5] and da Silva et al. [6], applied in capillary electrophoresis. Subsequently, Pumera and Wang [7] replaced the tubular electrode in C4D with two flat aluminum sheets and applied it in microchip electrophoresis. When the sample passed through the detection cell along the channel, the change of the local impedance caused the change of the alternating current on the pick-up electrode. After rectification, filtering, and amplification, the current on pick-up electrode was sampled and displayed as peaks [8,9].

At present, the common problems in microchip electrophoresis with C4D are high baseline background intensity, serious noise interference, poor detection sensitivity, and repeatability. These problems are mainly caused by stray capacitance and wall capacitance [10,11,12]. The wall capacitance can be reduced by integrated designs to control the thickness and material of the insulating layer [13,14,15]. Moreover, reasonably increasing the excitation frequency can also reduce the influence of wall capacitance. However, integrated designs are usually slightly more complex and increase the cost of the system. Meanwhile, increasing the operating frequency will increase the interference caused by stray capacitance. In severe cases, the interference even dominates the detector output, which has a fatal impact on the detection performance.

The method of arranging a ground electrode between the sensing electrodes to reduce the stray capacitance has been widely applied [8,16,17,18,19]. However, it is generally considered that its effect on reducing stray capacitance is limited. Based on the idea of ground electrode, Guijt et al. [20] built an integrated device design on a PCB to study the effect of insulation thickness on detection performance. Mahanadi et al. [21] added a pair of sensing electrodes to the traditional structure, forming a dual top-bottom geometry. This method doubled the coupling efficiency of the electrodes. However, the designs of microchip and electrodes are complicated, which may be the reason this method has not been adopted widely.

The other way is to counteract the adverse effects of stray capacitance and wall capacitance according to differential mode and impedance cancellation (circuit resonance) principle. Based on the principle of impedance cancellation, Shen et al. [22,23] obtained the minimum impedance with a low-impedance capacitively coupled contactless conductivity detector (LIC4D) at the resonance frequency. This method is achieved by connecting a piezoelectric quartz crystal (PQC, acts as an inductor) in series between the C4D cell and the excitation signal. Compared with traditional C4Ds, the signal-to-noise ratio of LIC4D was increased by more than 20 times. One problem is that the operating frequency of the actual inductor was difficult to adjust [24]. Based on differential mode, Laugere [25] introduced an integrated four-electrode design of the detector. Compared with the traditional bipolar configuration, the reported detector had a better signal-to-noise ratio in a wide frequency range, as low as 600 Hz. Georg Fercher [26] also adopted the similar method, and the obtained experimental result showed that the baseline intensity was greatly reduced, and the detection sensitivity was increased by several times. Xiao [27] and Stojkovic M et al. [28] introduced differential mode into capillary electrophoresis, which also improved the sensitivity of the system. Although many researchers have proposed solutions to counteract the adverse effects of stray capacitance, complex design requires complicated and strict fabrication processes and increases the cost of the system. An economical, easy-to-fabricate, sensitive C4D design for microchip electrophoresis is in demand, especially for the large-scale applications in environmental monitoring.

In this study, we described a planar grounded C4D (PG-C4D) based on PCB for microchip electrophoresis. The electrophoretic responses demonstrated that the PG-C4D had smaller background baseline intensity than traditional C4D, which can be attributed to the reduction in stray capacitance. Subsequently, the electrophoretic detection of three inorganic cations, K^+^, Na^+^, and Li^+^, proved that PG-C4D had better signal-to-noise ratio and repeatability. This simple manufactured, low-cost, and sensitive PG-C4D for microchip electrophoresis has great potential application in field applications.

## 2. Materials and Methods

### 2.1. Prototyping of Microfluidic Devices

**Figure 1:**
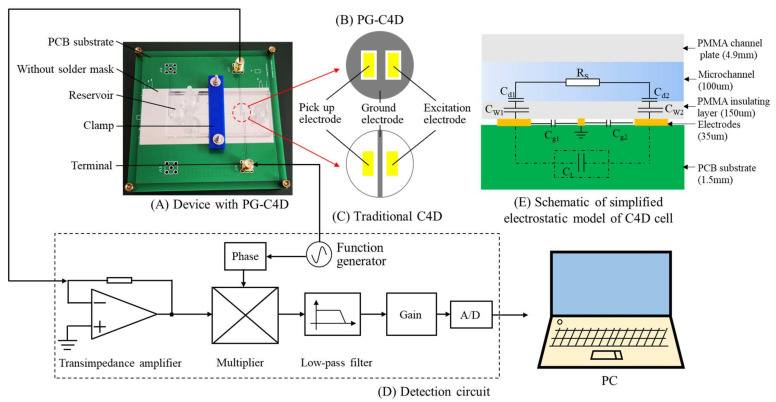
Schematic drawing of a microchip device with external C4D. (**A**) Device with PG-C4D (**B**) PG-C4D. (**C**) Traditional C4D. (**D**) Detection circuit. (**E**) Schematic of simplified electrostatic model of C4D cell.

The layout of the microchip device with PCB based PG-C4D is shown in Figure 1. The microchip was secured on the PCB based C4D using a clamp and two screws, as shown in Figure 1A. The C4D electrodes were fabricated on a commercially available PCB (Shenzhen JLC Electronics Co., Ltd., Shenzhen, China). The solder mask was removed to reduce the distance between the electrodes and the microchip. The C4D electrodes were composed of a pair of sensing electrodes (1 mm width, 2 mm length, and 35 μm thickness) and a ground electrode. The sensing electrodes were spaced by an 800 μm gap, optimized in the previous reported work from our group [29]. The difference between the PG-C4D and the traditional C4D was the ground electrode design. The ground electrode in PG-C4D surrounded the sensing electrodes, while the ground electrode in traditional C4D lay in the middle of the sensing electrode pairs with width of 200 μm, as shown in Figure 1B,C.

The microchips consisted of a 5 mm thick PMMA plate and a 150 μm thick PMMA film. PMMA plate was used as the microchannel layer. The PMMA film was used as the insulating layer. The microchip was fabricated using the following steps. First, the microchannels in the PMMA plate were fabricated by a CNC machine (X5 combo, SYIL MACHINE TOOLS Co., Ltd., Ningbo, China). The length of the injection and separation microchannels were 16 mm and 53 mm, respectively. Both microchannels had a square cross section of 100 μm × 100 μm. Secondly, the PMMA plate with microchannels was cleaned with purified water in an ultrasonic cleaner (SB-5200DT, NINGBO SCIENTZ BIOTECHNOLOGY Co., Ltd., Ningbo, China) three times, 10 min each time. Then, the PMMA plate along with the PMMA film were air plasma treated at 700 mTorr at high power (30 W) for 5 min (PDC-002, Harrick Plasma, Ithaca, NY, USA). Next, the PMMA plate with microchannels was aligned onto the PMMA film and thermally pressed at 105 °C under 0.3 MPa for 15 min, leading to a strong adhesion between the layers. Finally, the bases of plastic pipette tips were attached on the top of reservoirs on the PMMA plate, in order to connect the microchip with external devices. A device of microchip electrophoresis with PG-C4D based on PCB, CNC and thermal press packaging is easy to process and economical, which is favorable for commercialization.

### 2.2. Reagents and Electrophoretic Procedures

All reagents were of analytical grade and purchased from Macklin (Shanghai, China). The solutions were prepared using deionized water (resistivity 18.2 MΩ · cm) processed through a purification system (Cascada I, PALL, Beijing, China). Stock solutions of cations (K^+^, Na^+^, and Li^+^, 5 mM) were prepared from their corresponding chloride salts. The 60 mM stock solutions of L-histidine (His) and 2-(N-morpholino)-ethanesulfonic acid (MES) were prepared daily. A mixture of 20 mM MES and 20 mM His at pH 6.0 was used as running buffer, and was filtered through 0.22 um nylon syringe filters before use.

To improve the repeatability, prior to use, the microchannels were washed with 100 mM sodium hydroxide solution, deionized water, and running buffer for 15 min, respectively. The sample solution was prepared daily in running buffer, avoiding sample stacking. At the end of a working day, the microchips were rinsed with deionized water, to prevent clogging and contamination. Sample injection was performed electrokinetically using cross-injection method.

**Figure 2:**
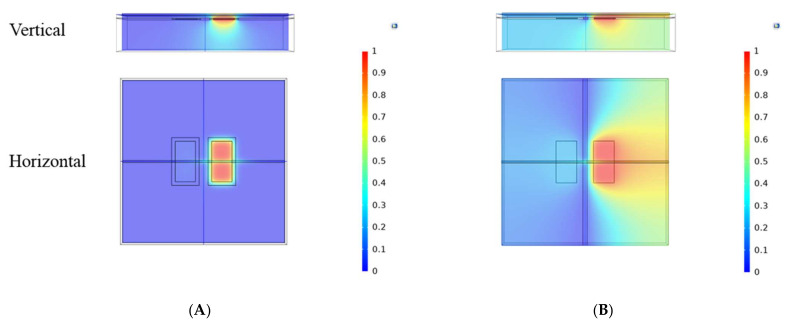
Simulated electric potential distribution of C4D cell, with both vertical and horizontal direction. A model (**A**) with PG-C4D feature and (**B**) with traditional C4D feature.

When the sample passed through the detection cell, the cell impedance would change, thus the sinusoidal current output of the PG-C4D would change. The I/V conversion was completed by a transimpedance amplifier (OPA656, Texas Instruments, Austin, TX, USA) with a feedback resistor of 2MΩ [30]. This voltage signal was then lock-in amplified [31], filtered and gained, and finally sampled by an acquisition unit as shown in Figure 2. The high-voltage modules and associated circuits used in the system have been described in the previous work [32]. The data acquisition was done by a data acquisition unit (MAX194, Maxim Integrated, San Jose, CA, USA) and a software written in LABVIEW (National Instruments, Austin, TX, USA). The software was also used to control the high voltage switching from injection to separation step.

### 2.3. Electrostatic Model and Simulation Settings of C4D

In order to obtain the influence of stray capacitance on solution conductivity detection, the equivalent circuit model was analyzed using finite element simulation. C4D with different ground electrode design adopted the electrical model shown in Figure 1E, which was simplified from an RC network proposed by da Silva and do Lago et al. [5,6]. C_L_ is the stray capacitance. C_W1_ and C_W2_ are the wall capacitance of sensing electrodes coupled to the microchip, which are equal to C_W_. C_g1_ and C_g2_ are the coupling capacitance between the sensing electrode and the ground electrode, which has negligible effect on the output current of the C4D [10]. The electric double layer C_d1_ and C_d2_ are very important in contact conductivity detection, but can be ignored in contactless conductivity detection [5]. Then the impedance of the C4D cell is defined as Z [11]. As shown in Equation (1), it is a function of C_W_, R_S_, and C_L_.

(1) $$Z=\frac{1}{j\omega C_{L}}//(R_{S}+\frac{2}{j\omega C_{W}}),$$

(2) $$\frac{1}{Z}=\;2j\pi {\mathrm{fC}}_{L}+\frac{1}{\frac{1}{j\pi fC_{W}}+R_{S}},$$

From Equation (1), R_S_ is connected in series with C_W1_ and C_W2_, and then connected in parallel with C_L_ to form a cell impedance. The reciprocal of the impedance, as shown in Equation (2), shows more clearly how the C_L_, C_W_, and R_S_ affect the cell impedance. C_L_ will cause additional and unwanted signals, which are not affected by the change in conductivity of solution. The unwanted effect of C_L_ increases with frequency, and severely impairs the detection performance of the system.

**Figure A1:**
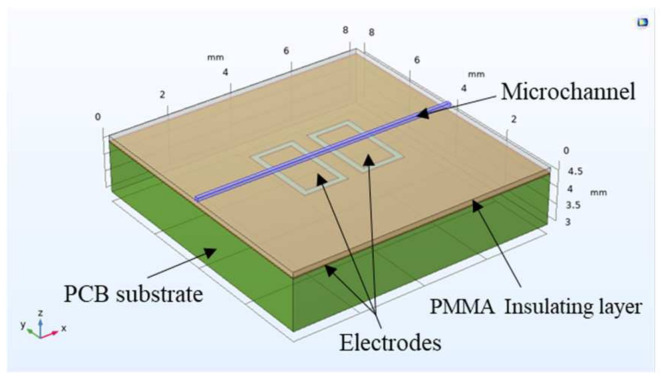
Three-dimensional model of the prototyped PG-C4D.

To study the stray capacitance generated by C4D with different ground electrode designs, a cell model was established by the Multiphysics simulation software COMSOL 5.6. The microchannel section area and electrode dimensions were set as mentioned above. To improve calculation efficiency, only the space near the sensing electrode is simulated. The three-dimensional model of the prototyped PG-C4D is shown in Appendix A, Figure A1. System default material parameters were adopted. Detailed information of the C4D is listed in Table 1.

## 3. Results and Discussion

### 3.1. Effect of Stray Capacitance on Cell Response

The effect of stray capacitance in both PG-C4D and traditional C4D was simulated and calculated, demonstrating that planar grounded electrode would greatly decrease the stray capacitance. The simulation result was then validated by baseline strength testing.

The cell equivalent circuit and physical simulation model of C4D were established as mentioned in Section 2.3. The electric field distributions of both PG-C4D and traditional C4Ds were obtained. Figure 2A,B show the calculated electric field intensity distributions in vertical and horizontal direction for both C4Ds. For the PG-C4D, the electric field was concentrated around the electrode to which the positive potential was applied. This is due to the presence of the planar grounded electrode. While in traditional C4D, potential distributed away from the electrode of which the positive potential was applied. Furthermore, the potential of the other electrode without positive potential was not 0 V. The potential distribution of the traditional C4D meant that there was charge accumulation on the other sensing electrode (the terminal where no potential was applied). This indicates that there is stray capacitance between the sensing electrodes in traditional C4D. The electric field distribution indicated that traditional C4D had larger stray capacitance than PG-C4D.

The stray capacitance was calculated based on the simulation result. Detailed calculation steps are shown in Appendix B [33]. The stray capacitance of the PG-C4D was calculated to be 11.698fF, while the stray capacitance of traditional C4D was calculated to be 38.81fF, which was more than three times of that of the PG-C4D.

**Figure 3:**
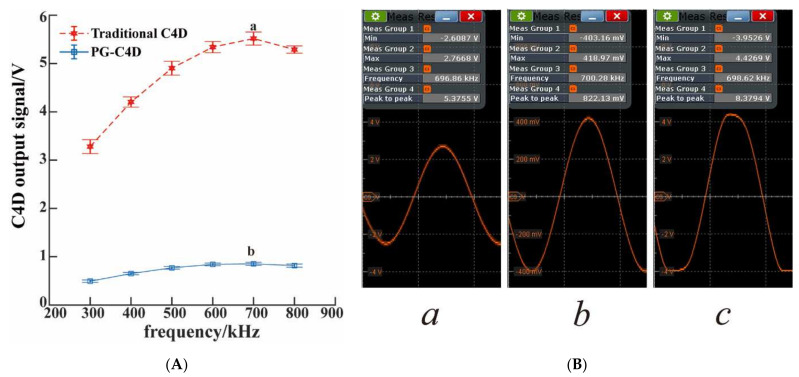
(**A**) C4D output signal. Red dashed line stands for the traditional C4D, and the blue continuous line stands for the PG-C4D. The error bars are the standard deviation of the measurements (*n* = 3). (**B**) Maximum output strength of the transimpedance amplifier. a: traditional C4D. b: PG-C4D. c: the excitation amplitude exceeded 10V in traditional C4D (distorted). Experimental conditions: channel filled with running buffer, oscilloscope filter cutoff frequency, 5 MHz.

To confirm the simulation result that the stray capacitance of PG-C4D was greatly reduced, the output signal (the average of baseline intensity) of the transimpedance amplifier was tested. It can be seen from Figure 3A that, compared to traditional C4D, PG-C4D effectively reduced the baseline intensity to one order lower than that of traditional C4D. The measurement of peak to peak (output) can avoid interference from other parts of the circuit, which is an indicator of whether the stray capacitance had reduced or not, as shown in Figure 3B. The baseline intensity result was on par with the previous reported C4D with the ground plane perpendicular to the channel [16]. The layout of the PG-C4D was simpler, more convenient, and suitable for large-scale production, and showed a good suppression effect on stray capacitance. In addition, the output signal of the transimpedance amplifier became distorted once the excitation amplitude exceeded 10 V. As shown in diagram c of Figure 3B, this distorted phenomenon indicated that the signal cannot keep sinusoidal signal, due to the bandwidth and output voltage limitation of the transimpedance amplifier (OPA656, ±5 V). This phenomenon also proved that the large stray capacitance in traditional C4D limited the applicable range of the excitation signal. The noise would also be more obvious under stronger excitation voltage. An effective method to introduce differential design would reduce the noise caused by high excitation voltage, but it would increase the cost of the system [20,26,28]. It could be concluded that PG-C4D can greatly reduce the interference current of the pick-up electrode due to the reduction of stray capacitance, and thus have better signal-to-noise ratio and detection sensitivity.

### 3.2. Performance Comparison

**Figure 4:**
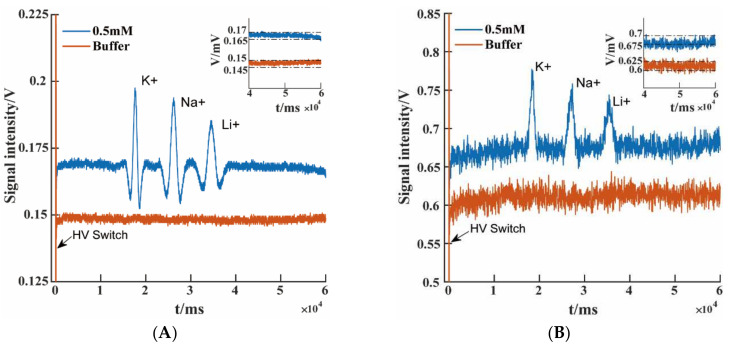
Raw electropherograms showing the separation of inorganic cations (K^+^, Na^+^, and Li^+^, 0.5 mM each). (**A**) PG-C4D and (**B**) traditional C4D. Injection voltage: 500 V; separation voltage: 1000 V. Running buffer: 20 mM MES/His, pH 6.0. Detection conditions: 700-kHz, 5Vpp.

The analytical performance of C4Ds with different ground electrodes was evaluated by inorganic cations detection experiment. The inorganic cations of potassium (K^+^), sodium (Na^+^), and lithium (Li^+^) in the mixed solution of each ionic compounds were used as detection objects. The raw electropherograms of the three inorganic cations are shown in Figure 4. The RSD values of migration times, peak areas, and peak heights were calculated to compare the detection results of the two ground electrode configurations. Table 2 shows the calculated results for the 0.5 mM concentration level for five consecutive injections (*n* = 5).

The high-voltage switching at the moment of sampling would cause the output of electronic devices to jump, as shown by the arrows marked in Figure 4A,B. The inset diagrams in Figure 4 showed the raw baseline signal noise, without any filtering algorithm, to better estimate the ability of PG-C4D to suppress noise. It could be seen from the inset diagram in Figure 4A that the noise amplitude of the separated sample and running buffer measured by PG-C4D was approximately 4 mV. The peak heights for K^+^, Na^+^, and Li^+^ were approximately 41.2 mV, 35.3 mV, and 23.7 mV, respectively. Here, the peak height was used to represent the peak intensity. The calculation process of the peak height was as follows. First, the signal was filtered, then peak function in MATLAB was applied to find the starting point, vertex, and end point of the peak. Finally, the peak height was obtained by subtracting the mean of the start and end points from the peak apex value. Signal-to-noise ratio of the peak height is greater than 3 for all the three ions. Though the concentrations of K^+^, Na^+^, and Li^+^ were all the same in the sample, peak intensities decrease in the order of K^+^, Na^+^, and Li^+^, which was related to the ionic conductivities of these ions [34]. The noise level in the traditional C4D is approximately 25 mV, as shown in the inset diagram in Figure 4B. The peak height for K^+^, Na^+^, and Li^+^ were about 94.5 mV, 75.7 mV, and 54.7 mV, respectively. The noise in the signal seriously affects the performance of the device with traditional C4D.

Compared with traditional C4D, the absolute peak height value of PG-C4D was reduced due to a slight loss of signal current. However, the signal-to-noise ratio was improved by 270%, 290%, and 270% for K^+^, Na^+^, and Li^+^, respectively. For traditional C4D, a large part of the current measured by the electronics was actually introduced by stray capacitance. The stray capacitance current caused larger output signal of the C4D, but it actually enhanced the noise proportion, which was harmful to the sensitivity of the system.

It could be seen from Table 2 that the RSD of migration time of K^+^, Na^+^, and Li^+^ increased slightly for both PG-C4D and traditional C4D, which was due to discrimination caused by electric injection [35,36]. The results in Table 2 demonstrated that the PG-C4D had better repeatability for quantitative analysis. For PG-C4D, the RSD for peak area was found to be 1.56% for K^+^, 1.57% for Na^+^, and 2.88% for Li^+^, and the RSD for peak height was found to be 0.43% for K^+^, 1.92% for Na^+^, and 3.42% for Li^+^. The results were on par with those obtained on referenced C4D with capillary electrophoresis [28] or complex top-bottom cell with precise shield of microchip electrophoresis [21].

### 3.3. Separation and Quantification of Cations

**Figure 5:**
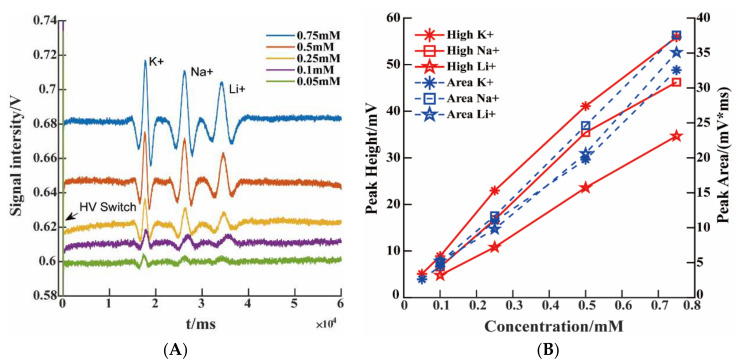
(**A**) An electropherogram of five concentrations. (**B**) Calibration curves of peak height sensitivity and peak area sensitivity of microchip electrophoresis with PG-C4D. Operational conditions were the same as in Figure 4.

**Figure A2:**
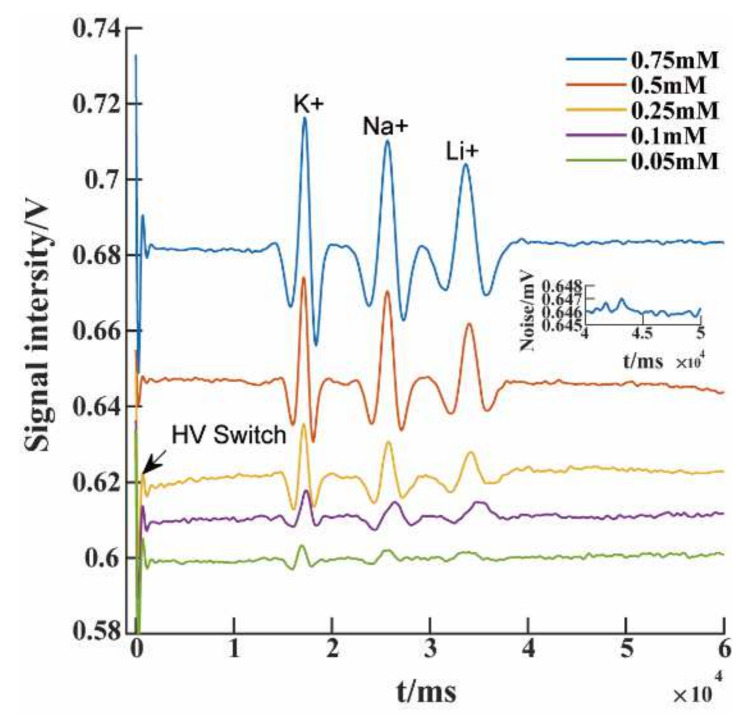
Signals filtered by Butterworth (cutoff frequency = 5 Hz), the inset diagram is the baseline noise fluctuation.

**Figure A3:**
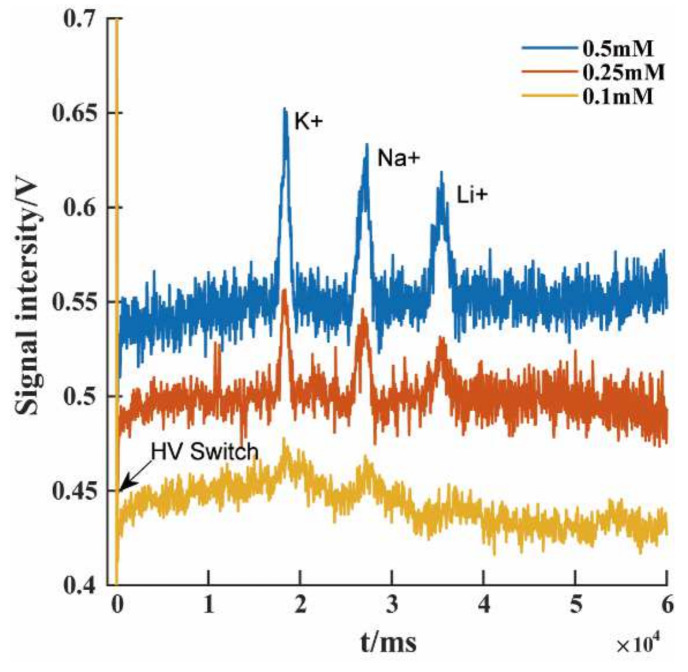
An electropherogram of three concentrations of device with traditional C4D. Operational conditions were the same as in Figure 5.

To demonstrate the quantitative performance of PG-C4D on cationic mixtures, five samples with different concentrations were tested. Figure 5A shows the raw electropherograms of the five concentration levels, under the optimal excitation signal conditions. The detection limits of K^+^, Na^+^, and Li^+^ cations were 0.05 mM, 0.1 mM, and 0.1 mM, respectively. The limit of detection was defined as the concentration at which the ratio of the peak height to the baseline noise obtained by filtering (S/N ≥ 3), as shown in Appendix C, Figure A2. The detection sensitivity here is relatively poor, compared with the C4D for microchip electrophoresis in a previous report [16]. However, under the same conditions, the detection limit of the proposed PG-C4D is much better than that of the traditional C4D. The detection limit of traditional C4D is around 0.25 mM, as shown in Appendix D, Figure A3. However, traditional C4D baseline noise fluctuates randomly and cannot be maintained at a stable level. The detection sensitivity of the proposed PG-C4D could be further improved by high voltage excitation signal [21,28] and a dedicated low-noise amplifier, but these devices would increase the cost of the total system [25,26].

The relation between the peak height/peak area and the concentration was investigated. The results showed that the peak height has higher concentration resolution. As shown in Figure 5B, the peak height/concentration correlation coefficients of K^+^, Na^+^, and Li^+^ were R^2^ = 0.9931, R^2^ = 0.9864, and R^2^ = 0.9986, respectively, and the corresponding sensitivities were 73.331 (K^+^), 62.213 (Na^+^), and 46.658 (Li^+^) mV/mM, respectively. The correlation coefficients of the peak area/concentration of K^+^, Na^+^, and Li^+^ were R^2^ = 0.995, R^2^ = 0.9991, and R^2^ = 0.9865, respectively, and the corresponding sensitivities were 41.694 (K^+^), 50.285 (Na^+^), and 46.145 (Li^+^) mV × ms/mM, respectively. These results are much better than those of the C4D with differential mode designed by Xiao [27] in capillary electrophoresis (such as, 2.2 × 10^−3^ (K^+^), 2.3 × 10^−3^ (Li^+^) mV/μM). The linear coefficients between the peak height against ion concentration and peak area against ion concentration of the same ion were almost the same. However, the sensitivity for peak height/concentration was higher than that of peak area/concentration. That is, peak height was a better indicator for concentration detection.

## 4. Conclusions

The reported PG-C4D had much lower stray capacitance than traditional C4D, thus the baseline intensity and noise amplitude of the detection cell were smaller, resulting in higher signal-to-noise ratio, detection sensitivity, and repeatability. Electrical field simulation and baseline strength test showed that the stray capacitance of PG-C4D was less than 1/3 of that of traditional C4D. The background baseline intensity of the PG-C4D was less than 1/6 of that of traditional C4D, and the noise amplitude of baseline was reduced from 25 mV to 4 mV. Electrophoretic detection experiments of inorganic cations showed that the RSD of migration time, peak area, and peak height were less than 0.3%, 3%, and 4%, respectively. The detection limits of inorganic K^+^, Na^+^, and Li^+^ are 0.05, 0.1, and 0.1 mM, respectively. Experimental results showed that the designed PG-C4D can be used in a wide range of excitation frequencies and amplitudes, which was especially favorable for systems with large signal excitation.

This paper proposes a new C4D device for microchip electrophoresis with low stray capacitance, high sensitivity, and high repeatability, which requires no special shielding and chip size and shape requirement. At the same time, the microchip and electrodes are produced independently, and the microchip is easily replaced through mechanical assembly. PCB, CNC machining, and hot-press packaging greatly reduce development costs, which are of great significance in device integration and large-scale applications. The designed PG-C4D shows great potential for microchip electrophoresis, including the detection of industrial wastewater, agricultural irrigation water nutrients, and environmental conditions.

## Figures and Tables

**Table 1:** Detailed information of the simulated C4D structure.

Title 1	Materials	Relative Permittivity	Size
Microchannel	Water	81	100 μm × 100 μm (cross-section)
PMMA insulating layer	PMMA	2.9	150 μm (thickness)
Sensing electrodes	Cu	1	2 mm × 1 mm × 35 μm (length × width × height)
PCB substrate	FR4	4.5	1.5 mm (thickness)

**Table 2:** Response Characteristics of PG-C4D and Traditional C4D (K^+^, Na^+^, and Li^+^, 0.5 mM each).

Ion ^1^	Indicator	PG-C4D (RSD)	Traditional C4D (RSD)
K^+^	Migration Time (ms)	0.15%	0.24%
	Area (mV × ms)	1.56%	2.48%
	Height (mV)	0.43%	4.74%
Na^+^	Migration Time (ms)	0.18%	0.39%
	Area (mV × ms)	1.57%	4.40%
	Height (mV)	1.94%	2.84%
Li^+^	Migration Time (ms)	0.23%	0.63%
	Area (mV × ms)	2.88%	9.46%
	Height (mV)	3.42%	6.28%

^1^ Data were filtered by Butterworth (cutoff frequency = 5 Hz) and obtained by trapezoidal numerical integration algorithm.

## Data Availability

Data is contained within the article.

## Appendix A

A model of the designed device geometry is shown in Figure A1. This device contains a 4-layer structure, the layers of which are microchannels, PMMA insulating layer, electrodes, and the PCB substrate, respectively.

## Appendix B

To calculate the capacitance, it was assumed that the sensing electrodes and the ground electrode form a mutual capacitance matrix. The capacitor matrix was composed of three capacitors, C_g1_, C_g2_, and C_L_, as shown in Figure 3. The calculation principle was slightly more complicated: here is a brief overview. Potentials were applied to the two sensing electrodes, respectively, and the corresponding charges on the electrodes were measured. The next step was to get the mutual capacitance matrix by converting the Maxwell capacitance matrix. COMSOL used stationary source sweep/or manual terminal sweep to set one terminal to 1 V and all other terminals to ground.

In the drop-down list of calculation results, capacitance matrix with different symbols could be obtained through global matrix calculation. In this way, we could obtain the mutual capacitance matrix between the three electrodes, and then obtained the stray capacitance coupled between the sensing electrodes.

## Appendix C

Figure A2 showed the filtered peaks and the fluctuation of baseline noise within 10 s. The filtered baseline noise was lower than 1.5 mV. It could be concluded that the filtering process can further improve the signal-to-noise ratio and it is beneficial for improving the detection limit of the system.

## Appendix D

Three samples of different concentrations were tested on the traditional C4D to compare the performance of the proposed PG-C4D with the traditional C4D, as shown in Figure A3. When the sample concentration is 0.5 mM, the peak heights for K^+^, Na^+^, and Li^+^ are about 94.5 mV, 75.7 mV, and 54.7 mV, respectively. When the sample concentration is 0.25 mM, the peak height for K^+^ is 52.6 mV, and Na^+^ 23.5 mV, while the peak height of Li^+^ cannot be identified due to excessive signal noise. When the sample concentration is 0.1 mM, none of the three ionic ions can be identified. These results also showed that the fluctuation amplitude of the baseline noise is unstable, which indicates that the traditional C4D had strong instability.
